# Developmental Programming of Fertility in Cattle—Is It a Cause for Concern?

**DOI:** 10.3390/ani12192654

**Published:** 2022-10-03

**Authors:** D. Claire Wathes

**Affiliations:** Department for Pathobiology and Population Sciences, Royal Veterinary College, Hatfield AL9 7TA, UK; dcwathes@rvc.ac.uk

**Keywords:** placenta, fetal programming, insulin, glucose, somatotropic axis, growth rate, ovary, immunity, puberty, age at first calving

## Abstract

**Simple Summary:**

Poor fertility is the main reason for premature culling of cattle. Dam factors and external variables can both influence how the calf fetus develops, known as fetal programming. Dam factors include age, parity, body condition, health and milk yield. External variables include nutrition and the environment during pregnancy. These all affect placental growth and the nutrient supply to the fetus, which in turn influence the size, shape and body composition of the calf. Postnatal growth rates, organ structure and immunity can all be affected. The impact on organs such as ovaries, liver, pancreas, lungs, spleen and thymus is dependent on the stage of pregnancy during which the fetal environment is sub-optimal. Regulatory systems which influence growth, metabolism and fertility can be permanently reprogrammed. Most changes affecting fertility are probably indirect. For example, calf health, the timing of puberty, age and size at first calving, and the ability to adapt to lactation after calving can all alter future reproductive potential. The size of these effects is hard to quantify due to practical difficulties in obtaining data over a sufficient time period. Nevertheless, there is compelling evidence that the fertility of some cows is compromised by events happening before their birth.

**Abstract:**

Cattle fertility remains sub-optimal despite recent improvements in genetic selection. The extent to which an individual heifer fulfils her genetic potential can be influenced by fetal programming during pregnancy. This paper reviews the evidence that a dam’s age, milk yield, health, nutrition and environment during pregnancy may programme permanent structural and physiological modifications in the fetus. These can alter the morphology and body composition of the calf, postnatal growth rates, organ structure, metabolic function, endocrine function and immunity. Potentially important organs which can be affected include the ovaries, liver, pancreas, lungs, spleen and thymus. Insulin/glucose homeostasis, the somatotropic axis and the hypothalamo-pituitary-adrenal axis can all be permanently reprogrammed by the pre-natal environment. These changes may act directly at the level of the ovary to influence fertility, but most actions are indirect. For example, calf health, the timing of puberty, the age and body structure at first calving, and the ability to balance milk production with metabolic health and fertility after calving can all have an impact on reproductive potential. Definitive experiments to quantify the extent to which any of these effects do alter fertility are particularly challenging in cattle, as individual animals and their management are both very variable and lifetime fertility takes many years to assess. Nevertheless, the evidence is compelling that the fertility of some animals is compromised by events happening before they are born. Calf phenotype at birth and their conception data as a nulliparous heifer should therefore both be assessed to avoid such animals being used as herd replacements.

## 1. Introduction

Many previous reviews have considered factors that affect dairy cow fertility in the period before, during and shortly after breeding. The objective of this review is to go back to the time of the cow’s own conception and examine the evidence suggesting that pre-natal programming may influence her fertility as an adult. Genotype is established at fertilization and also affects fertility, but this undoubtedly important aspect is outside the scope of this article. The extent to which genetic potential is fulfilled can, however, be affected by many influences to which the animal is exposed both pre- and post-natally. The studies reported here suggest that fetal programming during pregnancy can alter size, shape and body composition, postnatal growth rates, organ structure, metabolic function and immunity. Some of these changes may act directly on the ovaries but most probably act indirectly to alter reproductive potential, implying that fertility may become compromised by events happening during pregnancy before the calf is even born.

## 2. Fetal Origins of Adult Disease

### 2.1. Background

As described below, the development of the embryo and fetus are influenced by the dam’s age and size, her health and nutrition preceding and during pregnancy, fetal number and aspects of the external environment such as temperature. All of these factors and their interactions affect not only size at birth, but also how that neonate develops into adulthood. The epidemiologist David Barker first established that human babies that were born small due to poor intrauterine growth had an increased risk of developing cardiovascular disease during adult life [1]. His work suggested that the origins of some chronic diseases could lie in much earlier fetal responses to their intrauterine environment, a concept now widely referred to as the “fetal origins of adult disease”.

It is now well established that perturbations to the dam’s health, nutrition and environment during pregnancy may programme permanent structural and physiological modifications in the fetus. Growth occurs throughout both embryonic and fetal development through cell proliferation together with differentiation of the individual cells into the many different cell types required to form a viable newborn animal. The destiny of each cell is influenced by signals from both its neighbouring cells and the wider environment. If the supply of either nutrients or oxygen becomes inappropriate (restricted or excess), then internal signalling mechanisms operate to alter the rates of cell division, change the pathways of differentiation or cause apoptosis [2,3,4]. This can lead to disproportionate growth both within and between organs. Different parts of the body develop in sequence, with each tissue experiencing critical windows of rapid cell division during which they are particularly vulnerable to damage [5,6]. When nutrient supply is restricted, Hales and Barker [7] proposed that the fetus has some ability to reallocate available energy and nutrients to favour the development of organs critical for immediate survival (e.g., brain) over less essential organs and tissues including muscle and visceral organs such as the liver, pancreas, and spleen. These changes may help to sustain fetal development in utero by benefitting survival under continued conditions of malnutrition, but may permanently alter adult metabolism and immunity. This has particularly adverse consequences if the offspring is later exposed to an environment in which food supply is no longer limiting, with long-term implications for health and longevity [1,8,9]. As well as altering structure, some of these perturbations cause epigenetic changes leading to heritable modifications in gene expression by DNA methylation, histone modification or the influence of non-coding RNA causing gene activation or silencing [10,11].

### 2.2. Links to Fertility

Exposure of the mother to an adverse environment during pregnancy can not only influence her fetus directly, but may also have an impact on the germ cells that are forming during fetal development and may therefore also affect the fertility of the F2 generation [12]. In humans, rodents and sheep there is good evidence that adverse conditions experienced in utero can lead to programmed changes in the structure and function of the ovaries, the timing of puberty and alterations in menstrual/oestrous cycles, as reviewed by Yao et al. [13]. Relatively few studies have, however, tracked the long-term implications for fertility and most experiments involving deliberate manipulation of the embryo/fetal environment in livestock species have relatively short follow-up periods. Nevertheless, many of the changes reported could potentially alter fertility through a variety of mechanisms.

Establishing links between events during pregnancy and longer term postnatal development is particularly challenging in cattle. The nutritional supply to the animal itself encompasses not only the feed supplied at different stages of pregnancy, but also the dam’s body condition at the time, which affects the availability of additional nutrients through mobilisation of her body tissues [14,15]. Studies in sheep have demonstrated that fetal growth is sensitive to even a few days of maternal fasting [16], such as might occur during a brief illness such as mastitis [17]. Longer term nutrient restriction will have different effects. Fetuses appear most sensitive to maternal glycaemia but maternal protein deprivation is also important [18]. In addition to the main building blocks of proteins, carbohydrates and fats, many micronutrients including vitamins and minerals are absolutely essential for particular aspects of fetal development and their availability is not always easily recorded. Although outside the scope of this review, it is becoming clear that different nutrients (or their lack), can cause epigenetic changes through different mechanisms [19]. Perhaps counterintuitively, over-nourishing adolescents during pregnancy is also detrimental to fetal development as they prioritise the nutrient supply towards their own rapid growth. This occurs at the expense of the gravid uterus and reduces placental growth, utero-placental blood flow and nutrient delivery to the fetus [20].

A further complication is that most cows go through pregnancy while themselves lactating. The nutritional status of the dam is therefore impacted by her milk yield, which varies with maternal age and stage of lactation. This will in turn influence nutrient availability to the gravid uterus. Finally, the costs and practicalities of performing controlled studies and following up sufficient numbers of offspring are very considerable in cattle. Outcomes such as calf birthweight (BTW) (a full list of abbreviations used is provided in Appendix A) can be measured in the immediate postpartum period but measures of heifer fertility are not possible until at least 15 months later when breeding should have started. Cow fertility cannot be recorded accurately for several years as it needs to be assessed over successive pregnancies. By then many animals will have been culled for a wide variety of reasons, which may or may not have been influenced by the in utero environment [21]. The majority of studies in cattle in which a particular treatment has been applied to the pregnant dams are therefore too underpowered to have any realistic prospect of determining significant differences in offspring fertility. Nevertheless, some inferences can be made, in part by comparison with other species. Of these the sheep is probably the most relevant as it is also a ruminant and has a similar type of cotyledonary placenta.

### 2.3. Placental Development

Many effects on the fetus are mediated by changes in placental development. This is easier to study experimentally in sheep than in cattle, in part because pregnancy and lactation are not coincident. A review by Funston et al. [22] suggested, however, that the placentomes of sheep and cattle may respond differently to a period of undernutrition as growth of the placentomes slows during the second half of gestation in sheep but continues throughout pregnancy in cattle. Although most placental growth occurs in the first half of pregnancy, the majority of fetal growth in terms of mass increase takes place in the final third [23]. If the placenta does not reach a sufficient size initially, then it may become unable to deliver sufficient nutrients in late pregnancy when the demand is greatest. Severe undernutrition during pregnancy in sheep reduces placental weight, whereas less extreme reductions in the maternal diet or a low body condition score in early pregnancy both cause placental enlargement [24,25]. Maternal nutrient restriction in early to mid-pregnancy was associated with a reduction in placental weight at 80 days compared with placentae in ewes that had been adequately fed, but the situation had reversed by late gestation [26]. Such compensatory placental growth is thought to be an adaptation driven by the fetus to extract more nutrients from the mother and was shown to involve the insulin-like growth factor (IGF) system [27,28].

Placental vascularization may also be increased during undernutrition [29]. Conversely, Reynolds et al. [30] reviewed the evidence that maternal nutrition (both restriction and excess) and heat stress led to reductions in placental vascular development, which then caused fetal growth restriction. In partial accord with this, nutrient restriction of beef cows from day 30 to 125 of gestation reduced their placental weights, and the placentae remained smaller in late pregnancy at 250 days gestation despite subsequent re-alimentation, although fetal weight at this time point was not affected [31]. Redifer et al. [32] found that beef cow dams with a thin body condition score (BCS) produced lighter placentae. Some further studies in cattle have examined the morphology of expelled placentae obtained immediately after calving [32,33,34]. These all found significant positive relationships between the placental weight and the total cotyledonary surface area with the weight of the newborn calf.

### 2.4. Dam Parity and Milk Yield

Heifers are generally bred for the first time at about 60% of their mature body weight, long before they are fully grown [35]. They then calve for the first time at about 90% of mature weight, so growth must continue into their first lactation [36]. Immature mothers including both teenage humans [37] and adolescent sheep [20] partition nutrients preferentially towards their own continued growth at the expense of the developing fetus, associated with a reduction in placental size. In accord with this, van Eetvelde et al. [38] found that heifer placentae were smaller than those in mature cows, with a reduced cotyledonary weight and a smaller total cotyledonary surface area. In mares, it was shown that total blood flow to the uterus during the last 2 months of pregnancy was lower in those with zero to two previous foalings in comparison with animals that had experienced at least three previous foalings [39]. Maternal age and parity at first calving therefore have a significant effect on calf BTW. Holstein heifers produced by nulliparous (NP) or primiparous (PP) dams are consistently lighter in comparison with multiparous (MP) offspring [40,41,42]. In addition, calves born to Holstein heifers of <22 months of age had a BTW on average 2.75 kg less than those calving at over 22.5 months [43]. In contrast, Redifer et al. [32] reported that, although PP dams had lower placental and cotyledon weights than older MP dams, calf BTW was unaffected.

**Figure 1:**
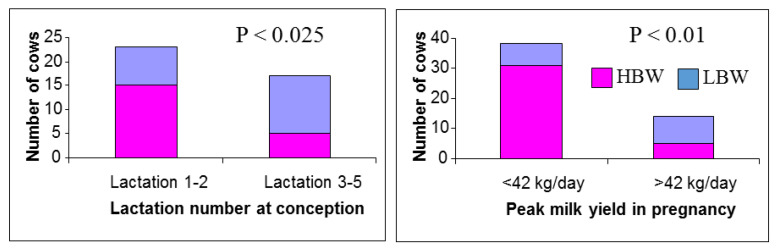
Relationship between maternal lactation number and peak milk yield during pregnancy on birthweight of Holstein-Friesian calves. Results are based on a study of 65 calves born concurrently on a single farm, which were divided into 3 groups: low birthweight (LBW, 32 ± 0.05 kg, *n* = 21), average birthweight (37 ± 0.03 kg, *n* = 22) and high birthweight (HBW, 42 ± 0.08 kg, *n* = 22). The number of LBW and HBW calves born in each dam category are shown. The proportions differed significantly according to dam lactation number and peak milk yield. Data from Swali and Wathes [44].

As already mentioned, MP cows are generally lactating for most of the time while pregnant and those conceiving soon after calving will be close to peak lactation during early gestation. Early embryo development therefore coincides with the competing demands of milk production, with the dams partitioning a significant proportion of their feed intake towards the mammary gland for milk synthesis during a period that is critical for placental growth. Although the nutrient requirements of the embryo are low in early pregnancy, their metabolic activity is high and this represents a critical period when many organs are starting to develop [4,6]. Swali and Wathes [44] investigated the effects of maternal parity and milk yield during pregnancy on growth of Holstein-Friesian calves. All the offspring from a single herd/breeding season were divided into three BTW groups. The low and high birthweight (LBW and HBW) groups averaged 32 kg vs. 42 kg and LBW calves were generally smaller in other measured indices (crown rump length (CRL), height, girth and ponderal index) between birth and 9 months. Retrospective analysis determined that the LBW calves were more likely to have older dams (lactations 3–5) with higher peak yields (>42 kg/day) (Figure 1). The impact of maternal age and milk yield on calf BTW was supported by a study by Kamal et al. [43], which found that cows in their second and third parities gave birth to calves that were estimated to be about 1 kg heavier when compared with calves born to older cows. Dams in which cumulative milk production between conception and drying off was either low (1400 to <5400 kg) or high (7200 to 11,600 kg) produced lighter calves than those born to average cows producing 6500 to <7200 kg.

Other studies have used a different approach to the same problem, by modelling large amounts of data generated through national databases to determine potential effects of maternal milk production during pregnancy. These focused mainly on offspring milk production but also included survival traits, which are affected by fertility. Berry et al. [45] interrogated the Irish national database for Holstein-Friesian cows to estimate the effect of maternal genetic variance on offspring performance. Greater dam milk yield pre-conception and during gestation was associated with small but significant reductions in offspring milk yield in the first and second lactation and reduced survival to second parity. Gonzalez-Recio et al. [46] reported that there was a negative effect on first lactation milk yield in a population of Spanish Holsteins cows that were conceived when their dams were lactating. This effect was significantly greater if the calves came from high yielding dams (>46 kg milk/day), in comparison with low yielding dams (20 kg milk/day). These results were partly supported by Banos et al. [47] in a population of UK Holstein-Friesian cows: they found that increasing maternal milk yield was associated with decreasing daughter milk production (measured as 305-d milk, fat, and protein yields) over lactations 1–3. These effects were significant but the differences were, however, too small to have practical relevance. Overall, these studies support the concept that high maternal milk production during pregnancy decreases energy partitioning to the fetus, and consequently drives fetal adaptations that affect their later performance capacity.

### 2.5. Climate

A series of studies have evaluated the effect of heat stress during pregnancy on calf development [48,49,50]. The main comparison has been between offspring of dams provided with evaporative cooling through their dry period at the end of pregnancy during the hottest time of year with offspring from uncooled cows. Heat stress reduced the dam’s dry matter intake, uterine blood flow and placental weight and cows calved, on average, 2 days earlier. This had the immediate outcome of reducing calf BTW by around 4 kg. Heat stressed calves remained lighter and shorter until 1 year of age but differences were no longer apparent at first calving. Another study investigated a seasonal effect on bovine placental development and found that cows calving in winter and spring in Belgium delivered placentae with smaller cotyledons than those calving in summer and autumn. The authors suggested that this may have been related to seasonal changes in food supply [38].

## 3. Impact of Fetal Programming on Size at Birth and Post-Natal Growth

### 3.1. Size at Birth

Much of the variation in size at birth in mammals is determined by the intrauterine environment rather than the fetal genome. This was clearly demonstrated in horses using extreme reciprocal size crosses between Shires and Shetlands [51] or using embryo transfers between ponies and Thoroughbreds [52]. These experiments both showed that a genetically large foal cannot reach its normal BTW when gestated in a uterus that is smaller than normal, and conversely that genetically small foals will be born heavier than usual if gestated in the uterus of a larger than normal mare. The direct heritability of calf BTW within breed of cattle was estimated at between 0.16 and 0.53 [36,53,54]. Maternal heritability of BTW in cattle is lower, with figures between 0.05 and 0.19 reported [53,54,55].

While BTW is clearly influenced by nutritional programming during pregnancy it is not, however, a very reliable indicator that it has occurred. The size of the effect is dependent on the stage of pregnancy, the duration of the nutritional treatment, and the body reserves, age and breed of the dam. For example, underfeeding either dairy or beef cattle during early gestation did not alter BTW [56,57,58], whereas BTW was reduced when nutrient restriction covered the first two thirds of gestation [59]. Two other studies found no effect of dietary restriction in late gestation on BTW, even though this is the time of maximum growth of the fetal calf [60,61]. However, BTW was reduced in both restrict fed pregnant NP heifers [62,63] and in PP heifers [64]. These results support the hypothesis that animals which are still growing partition available nutrients differently to mature cows, prioritising themselves over their fetus. Another issue with using BTW as an indicator of the adequacy of prenatal development is that it fails to take account of possible changes in body composition such as adiposity and skeletal development, which can also affect subsequent performance, as discussed in later sections.

### 3.2. Postnatal Growth

Studies in a variety of mammalian species have shown that pre-natal events can influence post-natal growth rates. There is an extensive literature on this topic regarding human babies [65]. In particular, most infants which are born small for gestational age (SGA) go on to demonstrate catch-up growth, defined as having a height velocity above the limits of normal for at least 1 year after a period of in utero growth retardation. It has become clear that the rate of postnatal growth is important in determining the outcome for that child. If it is too fast, then they are more likely to develop insulin resistance and type 2 diabetes as adults. If it is too slow, however, then adults will be of short stature and are also at higher risk of various diseases including diabetes and cardiovascular disease [66]. Similarly, low BTW rats which exhibited post-natal catch-up growth went on to develop obesity and diabetes, but this did not occur if the catch-up growth was prevented [67]. To further complicate matters, the outcome in humans appears related to morphology at birth (i.e., including height as well as weight) and different studies have used differing definitions and cut off points to define their SGA populations [68]. Interestingly with relation to fertility, some SGA children who experience rapid catch-up growth then go on to reach puberty at a younger age than their normal BTW peers, contributing to having a reduced adult stature [68]. Approximately 10–15% of all individuals born SGA do not, however, complete postnatal catch-up growth and remain of short stature [28]. Most such cases are not associated with growth hormone (GH) deficiency but rather exhibit varying degrees of resistance along the GH-IGF-insulin signalling pathway.

Results relating to birth size and subsequent catch-up growth in sheep and beef cattle need to be interpreted carefully as effects of maternal nutrition during pregnancy also influence the dam’s colostrum quality and milk production capacity. This will therefore affect the postnatal food supply and the growth rates of any calves that are being suckled. This is not, however, the case for dairy calves, which are usually removed from the dam within 24 h of calving and fed either milk replacer or milk from the dairy. Swedish dairy calves born with a LBW were shown to grow more rapidly than their heavier counterparts, demonstrating compensatory catch up-growth [40]. Similarly, the LBW offspring of PP Holstein-Friesian dairy cows had caught up in size with respect to weight, height and CRL within three months in comparison with MP offspring in the same herd [41]. In contrast, those calves born smaller within the MP offspring group remained smaller until at least 9 months of age in terms of weight and girth, although there was no longer a height difference [44]. Twin calves, which are born smaller than singletons, also grew more slowly [40]. In sheep, however, although twin lambs had lower BTW, this difference had disappeared by 1 year of age [69].

## 4. Influence of Fetal Programming on the Development of Specific Organs and Tissues

There are reports that many organs and tissues are affected by programming during pregnancy. Only those which are thought most likely to affect fertility have been included in this review. It should be noted that differences in outcomes between studies will be influenced by both the timing and severity of the treatment imposed relative to the main developmental window for each organ. There may also be sex differences in the responses. The focus here is on female fertility, so neither specific effects on the male reproductive system nor sex differences relating to male offspring have been reported.

### 4.1. Muscle and Adipose Tissue

When catch-up growth does occur, it may be associated with important alterations in body composition. The myofibre number is established during fetal development and muscle is not a priority tissue under circumstances of limited nutrient supply [22]. In cattle, the majority of muscle fibres form between 2 and 8 months of gestation [70], whereas in pigs their formation continues longer into the third trimester [71]. Hoffman et al. [72] measured gene expression in semitendinosus muscle of newborn lambs from ewes which had been either over- or under-nourished during pregnancy and found over-feeding altered expression of genes involved in regulating muscle protein synthesis and growth whereas nutrient restriction affected genes involved in muscle cell proliferation and signal transduction. Nutrient restriction during pregnancy may therefore limit postnatal increases in muscle growth [73]. In contrast, the major part of fetal adipogenesis and adipose tissue differentiation in sheep takes place during the final few weeks of gestation [74]. The nutritional programming effects on muscle versus adipose development are therefore both time and species dependent.

Both under- and over-nutrition of ewes during late pregnancy led to changes in fat deposition patterns in their six months old lambs, resulting from more abdominal (mesenteric or perirenal) rather than subcutaneous fat deposition [71]. Undernourishment also caused an increase in fibrosis in adipose tissue and the occurrence of a subpopulation of very small adipocytes. Other studies in both sheep [75] and beef heifers [63] similarly found that offspring born to dams which had been nutritionally restricted during pregnancy had increased internal fat when slaughtered. The lambs also tended to have proportionately reduced muscle weights. In pigs, the smallest fetuses born in a litter were shown to have a higher proportion of fat and collagen in their skeletal muscle than their larger littermates, which may be associated post-natally with reduced lean tissue growth and a predisposition for adiposity [76]. A metanalysis relating to human infants found that SGA babies were not only born with reduced muscle and fat, but in this case, their fat mass remained lower in follow-up investigations performed at up to 1 year post-natally [77]. This analysis did not, however, preclude there being later changes in adiposity. In support of this, Ibáñez et al. [78] showed that SGA children had similar height, weight, and body mass index to average BTW children at 2 years of age but they were more insulin sensitive at this stage and they subsequently gained more abdominal fat and less lean mass between 2 and 4 years of age.

### 4.2. Skeleton

Nutritional restriction in pregnant ewes resulted in reduced growth of long bones in late gestation [79] and dairy calves produced by PP dams were born significantly shorter by about 2 cm in height at withers in comparison with MP offspring [41]. This difference had been lost by 3 months, indicative of compensatory skeletal growth of the PP calves. Tygesen et al. [80] similarly reported that lambs born to ewes subjected to a 60% restricted feeding level for the last 6 weeks of pregnancy had a reduced femur weight at 5 months of age. However, the mean relative wall thickness of the bone was significantly increased, suggesting that compensatory growth mechanisms were disproportionate. Heasman et al. [81] found that CRL was significantly greater in lambs born to nutrient-restricted ewes and, in accord with this, Sullivan et al. [82] examined the effects of supplemental protein during pregnancy on composite beef heifers. More protein increased maternal IGF-1 and the dam’s IGF-1 concentrations in late pregnancy were negatively associated with the calf CRL. Another study in MP beef cows compared high (12%) or low (6%) crude protein (CP) diets fed during the second half of pregnancy [83]. The ratios of both body length and height to BTW were significantly greater in the low CP calves. All of these studies therefore imply that calves born with a longer than normal length to weight ratio have experienced growth retardation in utero. A study in horses investigated the long-term orthopaedic consequences of immaturity at birth in comparison with normal foals, based on 25 different anatomical measurements [84]. This found that horses with a history of either prematurity or dysmaturity developed proportionately longer in the body relative to their height, with shorter distal limbs in comparison with full-term horses of the same breed. In humans, the majority of SGA babies (85%) had caught up with normal birth size babies in terms of both weight and height by 2 years of age [65]. For those children in which catch-up growth had failed to occur by puberty, further bone lengthening was prevented by fusion of the epiphyseal growth plates, resulting in persistently short stature [65,66]. This cessation of long bone growth at puberty also applies in other species.

### 4.3. Ovaries

The fertility of female mammals is strongly influenced by the number and quality of oocytes formed in the fetal ovary, a number which is fixed before birth [85]. This review has focussed on the effects of nutritional programming on ovarian development in sheep and cattle: readers are referred to Yao et al. [13] for a review of experimental results derived from rodent models. Nutritional restriction of ewes in early pregnancy led to a reduction in fetal ovarian mass at day 50 and at day 65 (mid gestation) there were significantly fewer germ cells present in the underfed animals, indicating delayed germ cell maturation and onset of meiosis [86,87]. Lea et al. [88] also examined folliculogenesis in fetal ovaries from ewes undernourished over different periods during early and mid-pregnancy. They found that the treatment altered immunoexpression of genes involved in regulating both proliferation and apoptosis in the germ cells, granulosa cells and developing ovarian vasculature in a time-dependent manner. Another study reported that primordial follicles from fetal sheep ovaries collected on day 135 of gestation following a 60% nutrient restriction from day 50 of gestation onwards had a decreased cellular proliferation rate compared with those from fetal ovaries of adequately fed ewes [89]. Nwachukwu et al. [90] fed ewes low protein diets for the first half of gestation. Ovarian weight in the fetuses was again reduced compared with the controls, but in this case there were no apparent differences in germ cell development at mid gestation. In contrast, Osgerby et al. [79] reported that fetal ovaries were heavier in mid gestation (day 90) in offspring of ewes which were undernourished in early gestation. Overall, these experiments support a direct effect of maternal nutrition on fetal ovarian development, but the outcome is very time dependent.

Ovarian development in cattle fetuses begins at around 50–60 days gestation. The oocyte nests start breaking down to form primordial follicles at approximately day 80, although there is a period during which oocyte nests, primordial follicles and some developing follicles are all present [91]. Work on nutritional influences on ovarian development in cattle has produced similar results to those in sheep. Sullivan et al. [92] fed beef heifers different concentrate allocations during their first and second trimesters of pregnancy and followed up on the ovarian parameters in their female calves both pre- and post-pubertally at 5 and 23 months of age, respectively. Heifers switched from a low to a high concentrate diet had smaller antral ovarian follicles present at 5 months and lower densities of primordial and primary follicles and healthy antral follicles when killed at 23 months.

The number of antral follicles present in the ovary can be used as a proxy measurement for the size of the follicular reserve, which has previously been associated with oocyte quality [93]. The size of the reserve is also associated with circulating concentrations of anti-Mullerian hormone (AMH) and follicle-stimulating hormone (FSH). Some studies have shown that cows with lower follicular reserves have reduced fertility, as assessed by lower pregnancy rates, more services per conception (S/C) and longer calving intervals [94,95]. Two studies by Cushman et al. [96] both found a relationship between the size of the follicle reserve with calf BTW, with smaller born heifers having smaller ovaries, fewer antral follicles and a decreased pregnancy rate. Mossa et al. [57] showed that dietary restriction during early pregnancy in beef heifers resulted in offspring with a diminished ovarian reserve as indicated by a reduction in antral follicle count, assessed using ovarian ultrasonography between 7 and 86 weeks of age. This was accompanied by reduced concentrations of AMH and increased concentrations of FSH. It was reported by Tenley et al. [97] that heifer offspring of PP Angus beef cows had a smaller ovarian reserve that offspring of MP cows, containing fewer antral follicles assessed by ultrasonography, and with fewer primordial follicles in histological sections. Gobikrushanth et al. [98] did not, however, find any relationship between serum AMH concentrations and dairy cow fertility.

### 4.4. Gastrointestinal Tract and Lungs

Impaired intestinal development in late gestation was reported in ovine fetuses that were growth-retarded following experimental placental reduction [99]. In contrast, Greenwood and Bell [18] reported that the weights of stomach, small and large intestines were unrelated to lamb birth size at any given empty body weight measured during postnatal rearing. When ewes were fed a restricted diet of 70% compared with 100% of their estimated requirements from day 22 of gestation onwards, fetal weight was not altered in late gestation but the heart and pancreas were proportionately lighter [79]. A short but acute nutrient restriction in mid gestation did not, however, alter the weights of liver, gut, heart, kidneys, pancreas, adrenal glands, spleen or thymus [100]. McMillen and Robinson [101] reported that experimental restriction of placental growth caused a relative decrease in liver weight in late gestation, together with an increase in the hepatic expression of 11βHSD-1 mRNA, suggesting that there had been increased exposure to cortisol. Alexander [102] similarly reported that the liver was disproportionately small in newborn lambs with intra-uterine growth retardation (IUGR), while Clarke et al. [103] showed that the ratio of liver to brain weight was lower in light compared with heavy twin lambs. Hyatt et al. [104] demonstrated that lambs born to NP mothers were lighter at birth and had smaller livers: although their body weight had caught up by 1 month of age, their livers remained lighter. Furthermore, in another experiment, they showed that male lambs from underfed pregnant ewes still had significantly smaller livers when slaughtered as three year old adults [105]. Twin lamb fetuses also exhibited disproportionately lighter kidneys, thymus, liver and thyroid than singletons in late gestation (S. McMullen, J. C. Osgerby and D. C. Wathes, unpublished observations).

The liver plays a central role in the regulation of metabolism and energy supply and is also critical to the working of the somatotropic axis, as it synthesizes the majority of circulating IGF-1 and IGF-2 and their binding proteins (IGFBPs) [106]. This aspect is discussed below. Maternal undernutrition in rats also led to a reduction in growth of the fetal liver together with permanent re-programming of liver metabolism towards a ‘starved’ setting, including changes in activities of glucokinase and phosphoenolpyruvate carboxykinase. These are key enzymes regulating glycolysis and gluconeogenesis [107]. There is currently a lack of evidence concerning possible effects of fetal programming on hepatic development in cattle. If similar changes do occur to those reported in sheep and rats, then this is likely to have a major impact on their ability to respond appropriately to the metabolic challenges of lactation.

There is also evidence that fetal programming may adversely affect lung development, so making the offspring more susceptible to developing respiratory disease. Fetal lung growth is largely dependent on expansion by fluid secreted from the pulmonary epithelium [108]. An insufficient volume of amniotic fluid can cause exaggerated trunk flexion and increased pressure in the thoracic cavity, so limiting lung expansion [109,110,111]. Both fetal lung hypoplasia and prematurity can result in compromised airway and alveolar formation, with long-lasting effects on postnatal lung function [112,113]. A short period of acute undernutrition in mid gestation sheep, such as might occur during an illness, reduced thoracic girth and uterine fluid volume at day 90 and decreased fetal lung weights at days 90 and 135 [100]. Furthermore, in sheep, growth restriction during late gestation until term by embolizing the umbilico-placental vascular bed resulted in LBW lambs with reduced lung compliance [114]. In cattle, Long et al. [115] found that the lungs and trachea of steers slaughtered at 16 months of age whose dams were fed a restricted diet from 32 to 83 days of pregnancy were significantly lighter than those from dams on a moderate diet.

### 4.5. Immune System

Both the thymus and spleen are major immunogenic organs for which there is evidence of fetal programming that could impair postnatal immunity. T-cell formation takes place in the thymus and lymphopoiesis occurs in the spleen, which has a key role in immunoglobulin production, lymphocyte regulation and enhancing phagocytosis [116,117]. A maternal low protein diet during pregnancy in rats induced long-lasting alterations in adult male offspring in both thymic structure and lymphocyte maturation and selection processes [118] and disproportionately reduced the growth of the spleen [107]. This was supported by a study of IUGR human babies, which developed a disproportionately small thymus [119]. On the other hand, Alexander [101] reported that both thymus and spleen were disproportionately larger in IUGR newborn lambs. A study in a human population used ultrasound scanning to track thymic development over the first year of life [120]. This also showed that growth patterns typical of poor fetal nutrition, particularly in the first trimester, were associated with poor thymic development. Similarly, nutrient restriction in sheep during most of gestation proportionately reduced the size of the thymus in late gestation lambs [79] and was associated with changes in both structure and function [121]. Heat stressed cows also give birth to calves with a smaller thymus and spleen than found in calves under cooling conditions [122]. There is also evidence that passive transfer of immunoglobulins from colostrum is impaired in heat stressed calves, possibly due in part to earlier gut closure [48,50]. Other reported alterations in postnatal immune function in calves from heat-stressed compared to cooled dams were less total plasma protein, serum immunoglobulin, haematocrit and B-lymphocytes [49,123]. All of these changes could potentially make newborn calves more susceptible to infection.

### 4.6. Anogenital Distance

Anogenital distance (AGD) is a feature relating to reproductive tract development that can easily be measured externally. The AGD is about twice as long in males than females and there is experimental evidence linking this measurement to pre-natal steroid hormone exposure. Treatment of late pregnant rats with adrenocorticotropic hormone (ACTH) to increase corticosteroid concentrations in their offspring resulted in an increased AGD in females at birth, but adults showed normal cyclic reproductive function [124]. Testosterone treatment of pregnant rats also causes masculinization of the AGD in female pups [125]. In women, excess androgen exposure in utero is a key element in the development of polycystic ovary syndrome (PCOS) and mothers with PCOS were themselves shown to produce fetuses with an increased AGD in comparison to the general population [126].

## 5. Influence of Fetal Programming on Postnatal Metabolism and Endocrinology

### 5.1. Insulin and Glucose

Insulin produced by the pancreas is important for stimulating glucose uptake into insulin sensitive tissues such as skeletal muscle and adipose tissue. Glucose uptake by the placenta and mammary gland is not, however, regulated primarily by the insulin-sensitive glucose transporter GLUT4, so both these tissues can continue to take up glucose from the circulation in the absence of insulin stimulation [127,128]. Adult glucose intolerance is caused mainly by changes in tissue insulin sensitivity but there may also be associated defects in insulin secretion. Cows become more insulin resistant during lactation, reducing glucose uptake by peripheral tissues and so making more glucose available for milk production [129]. Alterations in the functional capacity of the pancreas may therefore have lifelong effects.

Studies in rats and mice have shown that the intrauterine nutritional environment can cause changes in the structure and function of the fetal pancreatic islets [130]. When ewes were fed a restricted diet of 70% compared with 100% of their estimated requirements from early gestation onwards, the pancreas was proportionately lighter with respect to bodyweight in late pregnancy [79]. Both over- and under-nutrition of ewes during pregnancy reduced the number of fetal pancreatic B-cells present in late gestation [131]. Another study reported that undernutrition of pregnant ewes caused an increase in pancreatic islet size of their fetuses, together with sex-specific changes in the DNA methylation patterns of pancreatic tissue in late gestation [132]. Nutritional deprivation during the final stages of pancreatic development reduced insulin responsiveness in sheep fetuses [133]. In cattle, modest nutritional restriction in late gestation reduced pancreatic weight. This was associated with lower plasma insulin concentrations in the fetus compared with the controls but plasma glucose and glucagon were similar. No differences in pancreatic endocrine cell number or localization were revealed following histological analysis [134].

In terms of postnatal effects, lambs from nutrient restricted ewes in several studies showed evidence of dysregulation in their glucose secretion, based on measurements of circulating concentrations of insulin and glucose together with intravenous glucose tolerance tests (GTT). Ford et al. [75] found that the insulin response to a GTT was lower at 36 weeks postnatally in lambs from restrict-fed ewes compared with control-fed ewes, while the area under the curve (AUC) for glucose was higher, indicating reduced tissue sensitivity to glucose. Two other studies found that small lambs at birth [135] or those derived from restricted or overfeeding of dams during pregnancy [136] had elevated insulin concentrations compared to their counterparts, in association with evidence of insulin resistance. Gardner et al. [69] compared glucose-insulin homeostasis in single and twin offspring of ewes that were nutritionally restricted in either early or late pregnancy. The lamb BTWs were similar between groups by one year of age but the AUCs for both glucose and insulin were greater in those lambs that had been nutrient restricted in late pregnancy. This was associated with increased adipose tissue mass and reduced adipose tissue expression of *GLUT4*. Another study compared pairs of twin lambs that had at least a 25% difference in their BTWs. Both insulin and glucose tolerance were greater at 1 and 6 months in the smaller BTW lambs [103]. Khanal and Nielsen [71] similarly found that a depression of insulin sensitivity and reduced pancreatic insulin secretory capacity persisted into adulthood in lambs with a history of late gestation undernutrition. These studies in sheep therefore provide strong evidence for impaired insulin sensitivity of peripheral tissues developing in juveniles and adults following in utero nutrient restriction, and this was also associated with an increased adipose tissue mass.

In cattle, Kamal et al. [137] undertook a retrospective cohort study to evaluate potential associations between dam characteristics during gestation and insulin traits in newborn (3-day old) Holstein calves. The basal insulin concentrations were higher in calves born to the cows that had a higher total milk production during their pregnancy and a longer dry period: their insulin sensitivity, estimated using a GTT, was lower. The acute insulin response was also lower in calves born to cows having a longer lactation length. These results indicate that the dam’s milk production during pregnancy can alter the metabolic functions of her calf. Another study in MP beef cows compared high or low CP diets (12% vs. 6% CP) fed during the second half of pregnancy [83]. Calves born from low protein dams were hyperglycaemic during their first 60 days but glucose concentrations then returned to similar levels to those of high protein calves until weaning at 6 months. As insulin levels remained similar in the low protein calves, this suggested that they were insulin resistant. Long et al. [56] found that nutrient restriction in late gestation given to NP 15 month old beef dams did not influence either the BTW or postnatal growth rates of their calves. In this study plasma concentrations of glucose decreased more rapidly after a GTT in the restrict fed group, showing faster clearance. Offspring of NP crossbred beef heifers experiencing mild nutritional restriction during the first trimester were similarly unchanged with respect to BTW and postnatal growth rates but in this study glucose metabolism was also unaffected [57]. Tao et al. [49] examined the effects of heat stress or cooling provided to dry cows in late gestation on the metabolic responses of their calves. Heat stressed calves were born lighter but had caught up in weight by weaning at 7 weeks. When tested the week after weaning they exhibited a similar insulin response but faster glucose clearance to both a GTT and insulin challenge. Based on work in sheep outlined above, Van Eetvelde and Opsomer [138] suggested that such changes to their glucose metabolism in cattle would be likely to carry a higher risk for cows becoming more obese and insulin resistant by first calving. There are, however, different responses depending on whether the nutritional challenges were experienced in early or late gestation.

### 5.2. Somatotropic Axis

The somatotropic axis, including growth hormone (GH), IGF-1 and IGF-2, and their various binding proteins and receptors, is a key regulator of growth and it also plays essential roles in many other functions including the immune and reproductive systems [139,140,141]. In the postpartum dairy cow a reduction in circulating IGF-1 at calving is mainly controlled by a decline in hepatic GH receptors (GHR), regulated by peripartum changes in energy balance, cortisol and insulin [106,142]. The fall in hepatic GHR also affects the circulating concentrations of insulin-like growth factor binding proteins (IGFBPs), which influence the half-life of IGFs in circulation. In particular, hepatic production of IGF-1, IGFBP3 and its associated acid labile subunit (ALS) all fall while IGFBP2 rises in cows with a worse energy balance status [106]. Once the circulating IGF-1 has declined to very low levels, it may take many weeks to recover [143].

There is strong evidence that the somatotropic system in the developing fetus is modulated by prenatal programming [18,79]. Nutrient restriction during late gestation led to smaller pituitary glands in heifer offspring, containing a reduced number of GH-positive somatotrophs [63]. Hepatic neonatal IGF-1 mRNA concentrations were shown to be affected by maternal age, health, nutrition and cortisol levels as well as being associated with the hepatic GHR concentrations in both lambs [144,145] and calves [146]. Growth-retarded lamb fetuses were characterised by very high circulating concentrations of GH but low concentrations of IGF-1 [18,147] and they had reduced hepatic expression of both IGF-1 and ALS in late gestation [148]. In accord with this, circulating IGF-1 and IGFBP3 in fetal plasma were reduced in lambs from undernourished ewes, but IGFBP2 levels increased [79,149,150]. Twin lamb fetuses were smaller than singletons in late gestation and this was associated with lower IGF-1 concentrations (S. McMullen, J. C. Osgerby and D. C. Wathes, unpublished observations). When the maternal nutrient restriction only occurred in the first half of gestation, the fetal hepatic IGF-1 and IGF2 mRNA concentrations had, however, recovered by term [151].

The fetal serum IGF-1 concentration was positively correlated to BTW in sheep [152] and to fetal weight, growth rate, CRL and hip height in crossbred beef heifers [153]. The influence of supplemental protein during gestation on maternal hormones and fetal growth was determined in composite beef heifers [58]. This study reported a significant positive correlation between the cow IGF-1 concentration in early gestation (indicative of her having a poor energy balance status) and her calf’s IGF-1 concentration at birth, while Maresca et al. [83] described lower calf IGF-1 concentration at birth after maternal protein intake restriction from mid-gestation to calving in MP beef cows. Hyatt et al. [145] followed the subsequent postnatal development of juvenile lambs from ewes experiencing maternal nutrient restriction between 28 and 80 days of gestation, the time of early liver development. Gene expression levels for both *IGF1* and *IGF2* mRNA were increased in the offspring of nutrient-restricted dams. Noya et al. [58] found that reduced maternal nutrition in early pregnancy resulted in lower IGF-1 concentrations in their beef calves at 2 and 3 months of age, although this relationship was only present in one of the two breeds studied.

These studies collectively suggest that the concentration of GH, GHR and therefore also IGF-1 during the critical first few weeks of life are strongly influenced by the pre-natal environment. These differences are likely caused by a lack of hepatic GHR in calves following undernutrition, similar to the situation experienced in postpartum cows in negative energy balance [106]. Small newborn lambs are born with high plasma GH but low IGF-1, and this situation persisted for several weeks. After this, IGF-1 concentrations increased to a greater extent in small than in normal BTW lambs, suggesting postnatal resetting of the somatotropic axis [18]. As the IGF-1 concentration is highly correlated with the growth rate [154,155], this will have practical implications for smaller born lambs and calves, potentially limiting their catch-up growth immediately after birth, at a time when they are particularly vulnerable to infection. In humans, failure of catch-up growth in some SGA babies has been attributed to varying degrees of resistance along the GH–IGF–insulin signalling axis rather than to GH deficiency [28].

### 5.3. Hypothalamic-Pituitary-Adrenal (HPA) Axis

Another endocrine system for which there is good evidence of fetal programming is the hypothalamic-pituitary-adrenal (HPA) axis [28,156]. A rise in fetal glucocorticoids is a key activator of labour. Prior to that, glucocorticoids are crucial for the maturation of structure and function of the lungs, intestine, and endocrine systems and their circulating concentration can be increased during pregnancy by a variety of maternal stressors, including under-nutrition [157,158] or heat stress [159]. In accord with this, Bloomfield et al. [160] showed that periconceptional or late gestation under-nutrition of sheep led to preterm birth by premature activation of the fetal HPA axis.

Gardner et al. [161] measured resting or stimulated HPA axis function in offspring following undernourishment of sheep in early gestation (days 1 to 30). Resting plasma cortisol in nutrient restricted offspring was similar to controls at 4 months of age but had increased significantly in the female nutrient restricted lambs by 12 months. In accord with this, Hawkins et al. [162] reported increased activity of the HPA axis in 3-month old lambs following modest maternal nutrient restriction of ewes in early gestation. Another experiment in sheep examined the effects of maternal nutrient restriction in either early or mid-gestation on postnatal HPA axis function. The ACTH and cortisol responses of lambs to an injection of corticotrophin-releasing hormone was tested at 2, 5.5, and 10 months of age. Although BTW was not affected, the lambs exposed to early undernutrition in utero had a greater AUC for ACTH and cortisol response at two months, and significantly higher basal cortisol levels at 5.5 months of age [163]. In contrast, Wallace et al. [164] investigated HPA function in lambs at 9, 18 and 24 months of age following prenatal growth restriction caused by either small placental size or maternal undernutrition. Both treatments resulted in the birth of smaller lambs but neither altered postnatal HPA function. These data suggest that some stressors experienced by pregnant dams will increase corticosteroid exposure of their fetuses. This may have long-term consequences through: (1) altered structure of various organs; (2) premature birth and/or (3) some resetting of the sensitivity of the HPA axis in adults. Differential responses between male and female offspring have also been noted but are not covered here.

### 5.4. Hypothalamic-Pituitary-Gonadal (HPG) Axis

Few studies in either cattle or sheep have examined potential effects of fetal programming directly on the hypothalamic-pituitary-gonadal (HPG) axis. However, Borwick et al. [165] failed to detect any differences in a number of measurements of HPG activity between lambs born to ewes on complete or restricted diets. These were assessed either pre-pubertally at around 7–8 months of age or post-pubertally at 18 months. Measurements included luteinizing hormone (LH) pulse frequency, LH pulse amplitude, mean plasma LH and FSH concentrations and LH responses to GnRH. There were also no differences in pituitary concentrations of *LHB*, *FSHB* or *GNRH1* mRNA at either age. Another study into the effects of maternal undernutrition on the HPG axis found no effect on LH and FSH responses to gonadotropin-releasing hormone (GnRH) challenge between the female lambs derived from control or restrict fed ewes when these were tested at both 2 and 5.5 months of age. The magnitude of the pre-ovulatory gonadotrophin surge was also not affected by maternal diet [166]. This evidence suggests that the effects found in the ovaries as described above are probably not mediated by the HPG axis.

## 6. Follow-Up Studies Investigating Fetal Programming and Fertility

The studies reported above provide strong evidence that fetal programming during pregnancy can alter organ structure, metabolic function and immunity. The follow-up periods are, however, usually quite short and few extend onwards to determine long-term effects on fertility. Apart from direct actions on the reproductive tract, many of the changes reported could potentially alter fertility through a variety of indirect mechanisms. For example, the liver and gastrointestinal tract are essential for normal metabolic function, poor lung development will increase the risk of developing respiratory disease, and the spleen and thymus are both central to immunity. If any of these malfunction in later life then cows will be more prone to developing metabolic or infectious diseases, which will in turn have a negative impact on their reproductive potential.

### 6.1. Dam Parity and Milk Yield

The influence of dam parity on offspring fertility remains uncertain as there are limited studies and these obtained conflicting results. Swali and Wathes [41] found that the offspring of PP dams from a single herd conceived more rapidly during their first service period as NP heifers than those of MP dams, while fertility in the first lactation was similar between the two groups. This was not, however, replicated in a later, larger study which measured the fertility of NP heifers according to the age of their dams. This found that the offspring of both the youngest and oldest dams needed more S/C than those from dams in parities 2–5 (parity 1, 1.6 ± 0.10 S/C (*n* = 111); parities 2–3, 1.3 ± 0.05 S/C (*n* = 223); parities 4–5, 1.2 ± 0.06 S/C (*n* = 99), parity > 5, 1.5 ± 0.12 S/C (*n* = 60), *p* = 0.005) (J. S. Brickell and D. C. Wathes, unpublished observations). A retrospective study by Akbarinejad et al. [42] reported that offspring of MP cows were significantly more fertile than those of NP heifers with respect to days to first service, first service conception rate, S/C and calving to conception intervals. The values for the PP offspring were intermediate with, for example, average calving to conception intervals of 142, 136 and 127 days for the NP, PP and MP offspring, respectively. Another study involving dairy cows investigated the effect of dam parity or the feeding system of the dam (either a high or low level of concentrates) on the reproductive performance of daughters as maiden heifers, but did not find any significant relationships [167].

We also analysed the fertility data of heifers being bred for the first time according to the milk yield of their dam during her pregnancy (Table 1). This showed that the daughters of average yielding dams (305 d milk yield 7500–10,000 kg) performed best, achieving a significantly lower age at first calving (AFC) by an average of 48 or 37 days, respectively, in comparison with daughters of dams producing either <7500 kg or >10,000 kg milk during that lactation.

### 6.2. Dam Nutrition

A series of experiments in sheep investigated the effects of maternal nutrition during pregnancy on aspects of reproductive performance of their lambs. Gunn et al. [168] compared three different nutritional regimes in Scottish Blackface ewes kept on hill pasture and followed up their progeny over three successive breeding seasons. Some dams received a nutritional supplement during either the last 100 days of pregnancy or the first 100 days of lactation. Lambs derived from the un-supplemented control ewes subsequently produced more single than multiple births in comparison with either of the supplemented groups. The authors suggested that this might be due to embryo losses as they detected no differences in ovulation rate. Rae et al. [169] also investigated the effects of maternal undernutrition during pregnancy on adult reproductive function. In this experiment the ovulation rate was, however, reduced significantly in those animals whose dams had received a low energy diet until day 95 of gestation but there were no differences in basal FSH or LH profiles or gonadotrophin responses to GnRH. This suggested that there was an effect on ovulation rate but that this was not due to changes in pituitary responsiveness. In accord with this, Kotsampasi et al. [166] found that maternal undernutrition during mid- to late gestation led to a reduction in both the number and size of corpora lutea in postpubertal ewe lambs. This was supported by Long et al. [170] who reported lower plasma progesterone levels in ewes from nutrient restricted mothers, with fewer of them going on to lamb themselves (1/7 restricted vs. 7/7 for the controls).

In cattle, Sullivan et al. [92] manipulated the CP intake given to beef heifers during the first and second trimesters of pregnancy and followed up on various reproductive parameters of their female calves. When slaughtered at 23 months, the heifers derived from dams with a low protein intake in the first trimester had lower densities of primordial, primary and healthy antral follicles. Corah et al. [62] investigated the effect of dietary energy restriction to PP beef heifers during the last trimester of pregnancy and showed that their heifer offspring took on average 19 days longer to reach puberty than the offspring of unrestricted cows. Other studies examined the effects of protein supplementation in late gestation to beef cattle kept on a dormant range on the subsequent growth and reproduction of heifer offspring. Martin et al. [61] found that this did not affect BTW or age at puberty of their calves but significantly more of the offspring of supplemented dams calved in the initial 21 days of their first calving season (77% vs. 49%) and the overall pregnancy rates for the two groups also differed (93% vs. 80%). A later study by the same group did, however, find that age at puberty was reduced in heifers from protein supplemented cows and there was again a trend towards higher pregnancy rates in the supplemented group [171]. Using a different approach in dairy cows, Banos et al. [47] showed that cows having a higher maternal BCS during gestation improved their daughter’s fertility in terms of non-return rate and number of S/C. Furthermore, taking account of overall maternal effects (dam age at first and second calving, BCS during first gestation, and milk yield) accounted for a significant proportion of the total phenotypic variance of daughter calving intervals and non-return rates, both key measures of fertility.

### 6.3. Dam Heat Stress

A comparison of heifer offspring whose dams did or did not receive cooling during heat stress in late gestation found that there were no differences between the two groups for age at first service (AFS) or AFC but the cooled group of heifers required significantly fewer S/C (2.0 vs. 2.5) [48,49]. Survival rates before puberty were considerably worse in the heat-stressed group (22.7% vs. 12.2%), so the offspring which had been most affected by the heat stress did not have any fertility data recorded. An adverse effect of heat stress during the dry period on daughter fertility was also reported by Kipp et al. [172]. They performed a large analysis of production and fertility data from German dairy herds over an 11-year period and related this to the temperature humidity index (THI) from the last 8 weeks of pregnancy. In terms of daughter fertility, a THI ≥ 50 during this period was associated with longer intervals from calving to first insemination and the non-return-rate at 56 days after service decreased with increasing THI, confirming that heat stress can have an effect across generations. The effect of climate during the first trimester of pregnancy on ovarian reserves at 16 months of age was investigated by Succu et al. [173] by comparing heifer offspring conceived during the summer (THI of 69) or winter (THI of 55). Both the AMH concentration and antral follicle count were lower in summer born compared with winter born heifers but this did not alter their subsequent fertility at first conception.

### 6.4. Calf Morphology

One potential outcome of fetal programming is low BTW. This has the advantage of being easily measurable although, as discussed above, it is not a particularly reliable guide that other important changes to the developing fetus may have occurred. Low BTW may or may not be followed by postnatal catch-up growth and it can also be associated with alterations in body proportions.

**Figure 2:**
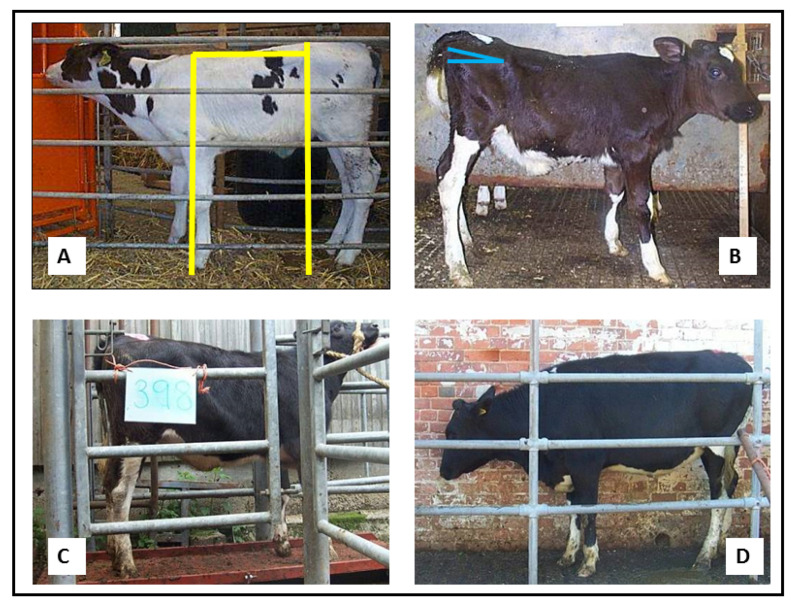
Photographs of dairy heifers born with high tuber ischia (pin bones), which gives rise to a low rump angle score. (**A**,**B**) Two calves at around 1 month of age. The same animal pictured in B is shown again at around 6 months (**C**) and 15 months (**D**) of age. Yellow lines in (**A**) illustrate that the hips are higher than the withers (the highest point of the thoracic spine). Blue lines in (**B**) show that the tuber ischium was higher than the tuber coxarum (hip bone). The rump angle is measured as the angle from the top of the tuber coxarum to the top of the tuber ischium.

**Figure 3:**
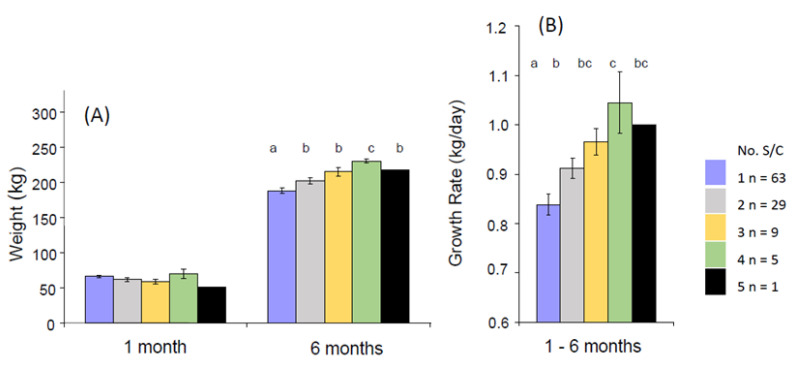
The relationship between the number of services required per conception (S/C) as nulliparous heifers and (**A**) the previous weight of the calves at 1 and 6 months and (**B**) their growth rate between 1 and 6 months. The numbers of animals in each category are given in the legend. Values are mean ± S.E.M. *p* < 0.05. a < b < c. (V. Wyse, J. S. Brickell and D. C. Wathes, unpublished observations).

One phenotype that we have observed was calves born taller at the hips than the withers (the highest point of the thoracic spine) and with a greater rump slope. This is defined as the angle between the height from the ground to the tuber coxarum and the tuber ischium (commonly referred to as the hip and pin bones, respectively, with the latter appearing as two raised areas on either side of the tail head [174]. Two individual pre-weaned calves with this phenotype are illustrated in Figure 2A,B. Calf B is shown again at both 6 months (2C) and 15 months (2D) of age. In each case, the sloping back remains visible with respect to the horizontal bars of the crush. Analysis of a population of 107 calves revealed that four of the six calves requiring ≥ 4 S/C as a NP heifer (3.7% of this population) started out with this phenotype, and all subsequently experienced a high growth rate in their first 6 months (Figure 3). A high rump angle is given a lower score on linear trait classification schemes for cattle. Wall et al. [175] examined the relationship between rump angle score and fertility in records from first lactation Holstein-Friesian cows. Animals with intermediate rump angle scores 4–7 had the shortest calving intervals, but differences were too small to achieve statistical significance. Van Eetvelde et al. [176] examined data from a population of cows achieving lifetime milk yields of >100,000 kg milk to determine features contributing to their longevity. They found that cows with a low rump angle score had decreased odds of reaching this lifetime threshold. Both these studies lend some support to the suggestion that having this phenotype at birth is indeed associated with reduced fertility. Rump angle slopes differ between breeds, being greater in Holstein Friesian than Jersey cattle [174], while some measurements of pelvic anatomy in heifers varied with season of birth [177] suggesting both genotypic and phenotypic influences. It is plausible that differential bone growth during pregnancy due to fetal programming may contribute to this phenotype, although it would be difficult to obtain direct experimental evidence to support this hypothesis.

**Figure 4:**
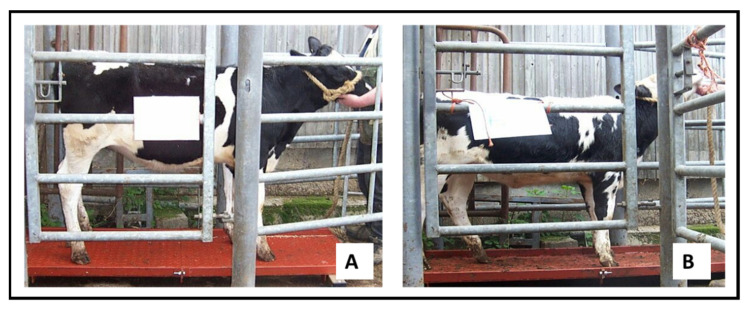
Two heifers measured at about 6 months of age. (**A**) is well proportioned, but (**B**) is short and fat.

Another disproportionate phenotype potentially caused by fetal programming is calves that are born short and light but which then experience significant postnatal weight gain, becoming short and fat. Figure 4 shows two heifers on the same farm being measured at around 6 months of age. Reference to the bars of the crush show that Heifer A is well grown, while B is much shorter.

Weight, adipose content and circulating IGF-1 in growing heifers all influence the timing of puberty, which in turn is a key determinant of when heifers can be bred and their likelihood of conception [35]. Delays in the timing of first conception inevitably affects the AFC. There is an increasingly strong body of evidence associating AFC with lifetime fertility and survival, with an AFC of around 23–24 months generally considered optimal [35,178]. Heifers can calve successfully at a younger age, but are then more likely to be insufficiently grown themselves. Being undersize at first calving, in particular with an inadequate frame size (reflective of skeletal growth) is associated with an increased risk of dystocia [179]. On the other hand, poor heifer fertility delays AFC and may result in overweight heifers calving for the first time. This can also cause calving difficulties and is associated with excessive and rapid loss of body condition in the early postpartum period [180]. Experiencing dystocia or being overweight at AFC can both reduce fertility of the primiparous cows [45,180].

The somatotropic axis plays an important role in determining the pre-pubertal growth rates in dairy calves. Both the GH secretion pattern [181] and the IGF-1 concentrations [154,155] are highly correlated with increases in both weight and height. Taylor et al. [182] found that plasma IGF-1 concentrations in dairy heifers measured at 6 months were positively correlated with their IGF-1 concentrations following first calving, whereas glucose concentrations had a negative correlation between these time periods. As well as regulating growth, IGF-1 also has multiple direct effects on the reproductive tract including ovaries, oviduct, uterus and placenta [27,183]. Associations of IGF-1 with fertility are discussed below in Section 6.7.

Swali et al. [181] found that weight and girth measurements of Holstein-Friesian calves at birth were highly positively correlated with the repeated size measurements at 3–9 months. Brickell et al. [184] investigated cohorts of replacement heifers born on 19 dairy farms. Higher measurements of weight and girth at 1, 6 and 15 months of age and increased skeletal growth (height at withers and CRL) at 6 and 15 months were all associated with a reduced AFB and AFC. The size range at 15 months was considerable (209–498 kg, *n* = 450). Sub-optimum growth of some heifers within each cohort was, therefore, established at an early age and resulted in animals reaching the start of breeding at an inadequate size. On the other hand, those with a greater girth measurement at 6 months or higher weight gains over the entire rearing period from 1 to 15 months required on average more S/C. These heifers presumably reached puberty earlier and were bred earlier but, although their initial conception rates were lower, they were still younger at first calving than heifers with poor growth rates [184]. In agreement with this, two previous studies which each compared feeding replacement dairy heifer diets to achieve average or accelerated growth rates both reported that those on the faster growth rates needed more S/C [185,186].

Perhaps surprisingly, Swali and Wathes [44] found that subsequent fertility in the LBW calves of MP dams appeared better than that of the HBW calves in their first lactation, with first service conception rates of 50% vs. 36% and average calving to conception intervals of 100 vs. 126 days, although the numbers per group (*n* = 17 and *n* = 18, respectively) were too few to achieve statistical significance. Twinning is another factor leading to low BTW calves. Giving birth to twins is known to negatively impact the subsequent fertility of the mother, and heifer calves with a male sibling become infertile freemartins [187]. There does not, however, seem to be data on the reproductive potential of pairs of female twins in cattle, although evidence from a large human demographic study of Danish twins born over a 20-year period did not find any evidence that being born a twin influenced their fertility [188].

Another study investigated the influence of bodyweight at first breeding in beef heifers on the reproductive performance of their offspring [189]. Angus-cross heifers were fed to reach either 55% (305 kg) or 65% (349 kg) of mature body weight at first breeding after which they all then received 100% of the recommended National Research Council requirements during the actual pregnancy. All the heifer offspring were subsequently fed to attain 65% of mature body weight at breeding. More of those heifers which were conceived in the 65% dams reached puberty at the start of the breeding season (58% vs. 49%) and became pregnant (92.8% vs. 87.2%). Furthermore, the difference in pregnancy rate was repeated in their second pregnancy (93.2% vs. 84.6%). This suggests that the more physically immature dams were partitioning more nutrients to themselves during their pregnancy and this had a long lasting effect on the fertility of their daughters.

### 6.5. Anogenital Distance

Anogenital distance is an accepted biomarker for prenatal androgenization and fertility in humans and rodents, so a series of studies have investigated its potential link to cattle fertility. Initial work in Canadian Holstein cows found that AGD was highly variable with a normal distribution, and the measurement was weakly associated with age and height. There was an inverse relationship between AGD and conception data in first- and second-parity cows but this was not evident in older animals [190]. Nulliparous heifers with a short AGD required fewer S/C (1.5 vs. 1.7) than those with a long AGD and so conceived on average 6 days earlier [191]. A subsequent validation study based on a larger population of 4709 cows confirmed that short vs. long AGD cows had a greater pregnancy rate to the first artificial insemination (AI) (36 vs. 30%), required fewer S/C (2.3 vs. 2.4), had fewer days open (137 vs. 142) and were more likely to be pregnant at both 150 and 250 days in milk [192]. Measurements of AGD were found to have a moderate heritability of 0.37 [193] and in general they were highly repeatable in the same animal over time, although measurements at birth did not reflect AGD at breeding age in heifers [194]. This association of AGD with fertility was replicated in a population of New Zealand Holstein-Friesian PP dairy cows [195] but not in Irish Holstein-Friesians with a range of parities [193]. In the latter study none of the reproductive variables measured differed significantly between AGD categories as measured at the time of breeding.

### 6.6. Metabolic Imbalance

Evidence presented above suggests that undernutrition in late fetal life can influence pancreatic insulin secretion directly and also cause differential insulin sensitivity between tissues, which will in turn alter their prioritization for glucose utilization. This will affect both the amount of adipose tissue deposition and the supply of energy going into the mammary gland for milk production. Van Eetvelde and Opsomer [138] reviewed ways in which fetal programming might predispose cows to the development of metabolic imbalance in early lactation and many previous reviews have highlighted the negative effects of metabolic imbalance on fertility (e.g., [196,197,198,199]).

Brickell et al. [154] found that dairy calves with increased growth rates during their first 6 months had higher glucose concentrations at 6 months. This probably reflects an association between energy intake and growth, as previously reported in both lambs and Holstein calves [136,200,201]. In terms of fertility, this may not be beneficial. NP heifers which failed to conceive at all tended to have greater weight and girth measurement at 15 months together with higher glucose and lower plasma urea concentrations. These measures might be associated with alterations in hepatic function, although this was not assessed directly. While reaching a greater size by the time of breeding was generally beneficial, this suggested that the largest size measurements could also be associated with poor fertility [184]. We also found a negative relationship between glucose concentrations at 6 months and those recorded by the same animals at the start of their first lactation [182]. Additionally, this study found that cows which experienced delayed ovulation in their first lactation had demonstrated insulin resistance at 6 months of age, as their glucose levels remained significantly higher after feeding despite a rise in insulin occurring at this time. Furthermore, dairy calves with higher glucose or beta-hydroxybutyrate concentrations when measured at 6 months were more likely to be culled in their second lactation. This was mainly due to infertility and was independent of yield [181]. These results suggest that some infertile heifers and those with suboptimal fertility were born with a low BTW and subsequently displayed signs of early catch-up growth, causing alterations in their fat distribution, insulin sensitivity, and subsequent reproductive potential.

### 6.7. Somatotropic Axis

**Figure 5:**
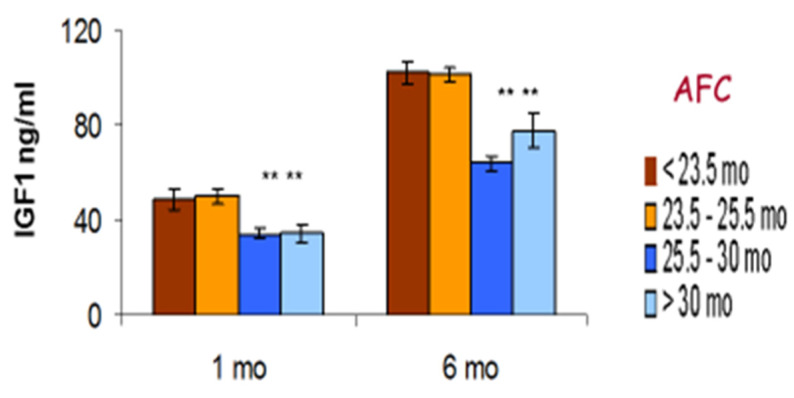
Measurements of circulating IGF-1 made at 1 and 6 months of age in dairy heifers according to their subsequent age at first calving (AFC). Data are from [182]. AFC < 23.5 months, *n* = 56; 23.5–25.5 months, *n* = 122; 25.5–30 months, *n* = 163 and >30 months, *n* = 36. ** *p* < 0.01 in comparison with <23.5 and 23.5–25.5 months.

There is a well established relationship between circulating concentration of IGF-1 in the early postpartum period and cow fertility (e.g., [143,202,203]). These studies show that cows which experience low concentrations of IGF-1 in the immediate postpartum period take longer to conceive and are more likely to be culled as infertile. The low IGF-1 concentration is caused by a major down-regulation of hepatic GHR and the cows also become more insulin resistant [106,204]. A study by Taylor et al. [205] determined whether any differences in the somatotropic axis in juvenile Holstein-Friesian dairy heifers assessed at 6 months of age were predictive of the timing of puberty or fertility in the first lactation. These were both monitored using progesterone profiles and puberty was reached at approximately 9 months of age. The BCS at puberty was positively correlated to the body weight and non-esterified fatty acid concentrations measured at 6 months and negatively related to the GH secretion profile and to circulating glucose. The animals which experienced delayed ovulation following calving had the lowest IGF-1 concentrations at 6 months, showed no decrease in circulating glucose concentrations following a postprandial rise in insulin and tended to have a higher GH pulse amplitude during fasting. Those prepubertal heifers calves that later developed a persistent corpus luteum following calving had the highest IGF-1 concentrations and a significantly larger GH pulse amplitude and pulse area than normal profile animals during the fed period. These results indicated that differences in the somatotropic axis of pre-pubertal calves were indeed reflected in altered reproductive function as PP cows. Later work on another cohort of dairy calves found that those with low IGF-1 concentrations at 6 months of age were less likely to achieve a first lactation at all, as they either aborted their first pregnancy or were culled after experiencing major peripartum difficulties [181]. We also found that dairy heifers which went on to have a delayed AFC were already distinguishable from their peers by having lower IGF-1 concentrations at each of 1, 6 and 15 months of age (Figure 5) [184].

### 6.8. Disease

A large body of work has investigated the long-term effects of dam morbidity during pregnancy on her offspring. This has mainly focussed on a wide variety of infectious diseases (viral, bacterial, protozoan, fungal) which can cause placentitis and/or be transmitted to the developing fetus, causing abortion, developmental abnormalities or postnatal infection [206]. Other infectious agents may cause the dam to become pyrexic, hypoxic or endotoxaemic, all clinical conditions with potentially adverse effects on the developing fetus. These are undoubtedly important but are not generally considered within the scope of fetal programming. Nevertheless, some diseases such as mastitis are very common in dairy cows [207]. Both clinical and subclinical mastitis alter the inflammatory status of the dam [208] and clinical mastitis will also cause a loss of appetite and reduced dry matter intake [17]. Gonzalez-Recio et al. [46] showed that cows derived from embryos which developed during episodes of maternal mastitis went on to have a shorter productive lifespan by 211 days on average in comparison with offspring of dams not so affected. This warrants further investigation.

The evidence reviewed above also indicates that fetal programming caused by suboptimal dam nutrition can affect the development of the thymus, spleen and lungs, all of which might be expected to put calves at greater risk of catching enteric and bovine respiratory disease (BRD), particularly during the pre-weaning period. Some studies have therefore investigated the relationship between calf BTW and disease risk. Corah et al. [62] and Windeyer et al. [209] both found that LBW was a risk factor for neonatal diarrhoea in beef and dairy calves, respectively, but it was not associated with BRD. Calves with a LBW were also reported to develop diarrhoea earlier post-natally than those of average BTW [210]. McCorquodale et al. [211] reported higher mortality rates in LBW dairy calves. Other studies did not, however, find that LBW was a significant risk factor for either diarrhoea or BRD in dairy heifer calves [212,213].

Overall, the relationships between BTW and calfhood disease are not very strong. One possible explanation for this could be that the critical windows of development which most affect the intestines, lungs and immune system do not necessarily result in measurable differences in BTW. Much stronger associations have, however, been found between morbidity and circulating IGF-1. Johnson et al. [155] showed that calves with a higher circulating concentration of IGF-1 in their first week of life subsequently had a reduced risk of developing either BRD or umbilical disease pre-weaning. Low IGF-1 in pre-weaned dairy calves was also a good predictor for calf mortality [214]. The IGF-1 concentration in pre-weaned calves is strongly influenced by nutrition, in particular it increases with the amount of milk fed [155]. This difference in IGF-1 according to diet does not, however, become properly established until the calves are about 3 weeks old [215]. This means that the circulating IGF-1 in the first two weeks is mainly influenced by pre-natal events. Having a low IGF-1 concentration at birth is therefore likely to be an important risk factor in determining the likelihood that calves become ill in their first few weeks of post-natal life. This would then set up a vicious cycle, as sick calves experience reduced growth rates [155,216], in part due to reduced feed intakes [217] and also because mounting an immune response is energetically demanding [218]. In terms of subsequent fertility, BRD and increased use of antibiotics (implying higher disease) in calves have been associated with a reduced risk of becoming pregnant [219], delayed AFC [220,221], increased odds of not completing their first lactation [222] and longer calving intervals in mature cows [223].

## 7. Epigenetic Modifications in Sperm

Although relatively little is known about this topic, it deserves a mention in the context of cattle fertility. Epigenetic modifications have been detected in bull sperm, which gave rise to preimplantation embryos with significant differences in their transcriptome (reviewed by [19]). This potentially allows transmission of traits influenced by epigenetic modification though the paternal germ line. Paternal nutrition causing epigenetic alterations in sperm may therefore have carry-over effects on their offspring, as reviewed by Dahlen et al. [224]. Evidence for this originally came from a human epidemiological study showing increased adiposity in the children of males whose pregnant mothers underwent severe undernutrition during the Dutch famine in 1944–1945, compared with controls not affected by the famine [225]. The possibility of paternally induced transgenerational reprogramming of metabolic traits associated with epigenetic modifications has since been confirmed in rodent models [226].

## 8. Assisted Reproductive Technologies

Another topic not already covered is the widespread use in cattle of assisted reproductive technologies (ART), including AI and embryo transfer (ET). In humans, there is evidence that ART can affect infant BTW and glucose metabolism, effects attributed to epigenetic changes in the early conceptus [10]. This is, however, a complicated issue as embryonic DNA undergoes demethylation and de novo remethylation between fertilization and the blastocyst stage, a process that organizes a fixed tissue-specific expression pattern in the genome. This is regulated differently between the male and female genomes and may be modulated by the maternal nutrient environment at the time [11]. One study found that the normal pattern of DNA demethylation and remethylation of the zygote was perturbed when heat-stressed spermatozoa were used [227]. This is a potential issue during AI as straws of semen may increase in temperature if there are any delays between removal from storage in liquid nitrogen and insemination.

With respect to fertility in cattle, one study compared the fertility of heifer offspring produced by AI or ET using either fresh (ET-F) or vitrified (ET-V) in vitro produced embryos [228]. No differences were detected in AFS, percentage pregnant at first service or AFC between these three groups. There were, however, trends for the ET-V group to perform worse than the ET-F group (AFS 396 v 386 days, *p* = 0.06); AFC 698 v 663 days, *p* = 0.1). These results need to be interpreted with caution as different bulls were used between groups and there were only 38–43 heifers per group. Differences recorded around birth (gestation length, calving induction, calving difficulty) could also have been influential in this study. Another larger retrospective analysis compared the fertility of offspring arising from in vitro embryo production (IVP, *n* = 2736) with offspring from multiple-ovulation embryo transfer (MOET, *n* = 3436). These techniques were both performed in the same facility over a 10-year period using animals balanced for genetic merit for breeding values. The IVP embryos were subsequently slightly more fertile as heifers than the MOET group, needing 1.9 vs. 2.0 straws to become pregnant, and with a 56 day nonreturn rate of 61.7% vs. 59.6% [229]. A similar study compared IVP and MOET produced offspring but focussed instead on male calves. Bioinformatic analysis of tissue samples obtained at 3 month of age showed differences in pathways involved in hypothalamic GnRH secretion, pituitary GnRH signaling and testicular steroidogenesis, suggesting earlier activation of the hypothalamus-pituitary-testicular axis in IVP compared to MOET produced animals [230]. There were also differences in expression of genes involved in energy regulation in liver and muscle samples obtained from the same animals [231].

## 9. Conclusions

Efforts by breeders to reverse negative trends in cow fertility caused by the previous overemphasis on production traits have led to small improvements in recent years. This follows greater inclusion of fertility and survival traits in genetic selection indices [232,233] and the increasing use of genomic selection for replacement heifers [234,235]. Poor fertility is, however, still a major concern and remains the main reason for involuntary culling in the dairy industry [236]. The evidence reviewed above provides substantial support for the concept of fetal origins of adult disease by showing that early life events can influence calf phenotype at birth and that this has the potential to influence fertility. This in turn will affect the extent to which an individual heifer can fulfil her genetic potential. We are, however, dealing with both dairy and beef animals of various breeds which are kept in many different environments and are managed very differently between farms. Much of the available literature is based on small numbers of animals kept on a single farm in which a specific treatment was imposed on the dam during her pregnancy. Other studies have used an epidemiological approach based on the animal’s phenotype at birth. Neither approach is ideal for measuring subsequent fertility: the studies are often underpowered and rarely undertake long-term follow-up or account for genetic differences between animals. Potentially important risk factors such as details of the dam’s diet, health and age may not be readily available. In practice the experiences of almost every cow/calf combination during pregnancy are unique, so it is unsurprising that conclusions vary between studies. The extent to which fetal programming does reduce fertility is therefore likely to be underestimated and it remains difficult to quantify its impact.

Nevertheless, some generalisations can be made. In practice, calves that are more likely to have problems with fertility can be identified before they are bred based on their appearance, size measurements (including height as well as weight) and growth rates. Taking a side view photograph of each pre-weaned heifer in her first week of life is a quick and simple action in dairy herds and this can be stored alongside other records of growth rates and health. This information should then be considered along with genotype when deciding which animals to use as herd replacements so that heifers with a poor phenotype can be avoided. Rearing unsuitable heifers to breeding age carries a significant cost as these animals are unlikely to ever become profitable [237]. Not all problems are, however, visible externally as fetal programming can also cause potentially important changes to internal organs, metabolism and endocrine systems. A second assessment should therefore be made after heifers have received two inseminations or at the end of the chosen breeding period in seasonal herds. Poor fertility at this stage is very likely to be repeated after calving, so again such animals should not be used as herd replacements.

**Figure 6:**
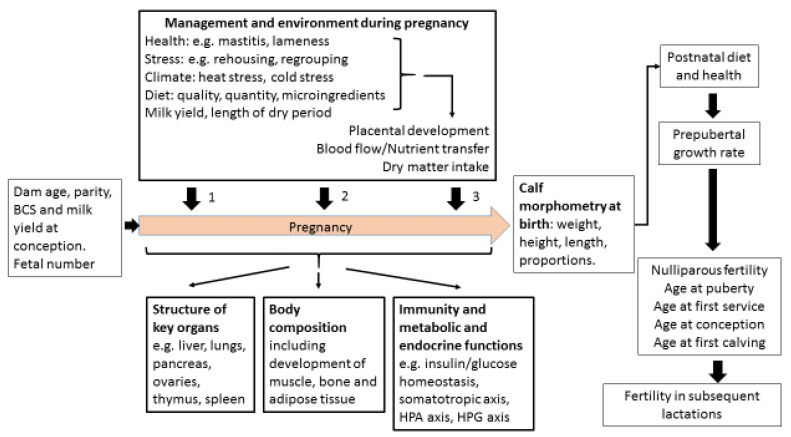
Schematic showing factors which may lead to fetal programming during pregnancy. Dam factors and fetal number are initially important. The dam is then exposed to a variety of management and environmental factors which can affect the developing embryo/fetus either directly or indirectly though changes in placental development, nutrient transfer and dry matter intake. The influence of the external factors will depend on both their severity/duration and also the stage of pregnancy when they occur (first, second and/or third trimesters). Depending on timing, they can affect organ structure, body composition and a variety of immune, metabolic and endocrine functions. Major changes in body composition will be evident at birth, whereas many of the other influences will be invisible but can, nevertheless, affect subsequent growth and health. These in turn affect the timing of puberty, so determining when heifers can be bred. Nulliparous fertility determines the age at first calving. Both the age and physical condition at which heifers calve for the first time then have a major influence on their subsequent fertility. Note that during both pregnancy and postnatal development over- as well as under-nutrition can have detrimental effects, particularly if there is a mismatch between the pre- and post-natal environments.

**Figure 7:**
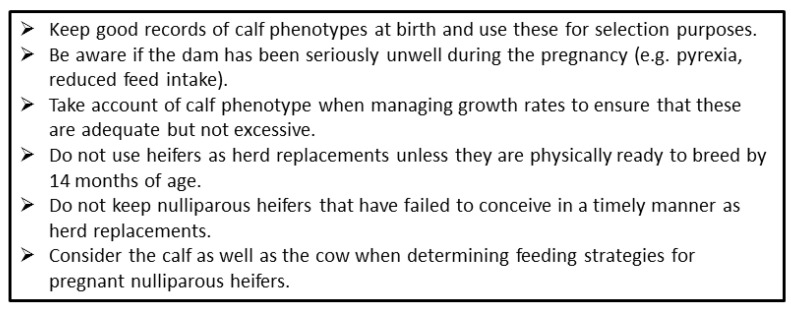
Key recommendations to farmers.

A greater awareness is also needed that any perturbations which affected the pregnant dams could have a permanent adverse effect on their developing calves. These could include a period of inappropriate nutrition (under- or over-feeding), adverse weather, or an episode of infectious or metabolic disease. Their impact will, however, be modified by factors such as their duration, stage of pregnancy, age of dam, milk yield, body condition etc, all of which will interact with each other. For example, NP heifers are likely to be the ones most greatly influenced by a period of either under- or over-nutrition during pregnancy. These factors and some of their possible interactions are illustrated in Figure 6 and some key recommendations for farmers are summarized in Figure 7.

## Figures and Tables

**Table 1:** Fertility of nulliparous dairy heifers according to the milk yields (mean ± SEM) of their dams during the pregnancy in which they were conceived.

Dam Previous 305 d Milk Yield (kg)	<7500	7500–10,000	>10,000	*p* ^1^
*n*	98	166	85	
Age at first service (d)	536 ± 18.3 ^a^	461 ± 6.8 ^b^	500 ± 12.4 ^a^	0.000
Age at conception (d)	558 ± 16.5 ^a^	513 ± 9.0 ^b^	541 ± 17.5 ^a^	0.034
Age at first calving (d)	839 ± 16.5 ^a^	791 ± 9.0 ^b^	828 ± 17.9 ^a^	0.018

^1^ Within rows, a > b.

## Data Availability

Not applicable.

## Appendix A. List of Abbreviations Used in the Text

**ACTH**, adrenocorticotropic hormone; **AFC**, age at first calving; **AFS**, age at first service; **AGD**, anogenital distance; **ALS**, acid labile subunit; **AMH**, anti-Mullerian hormone; **ART**, assisted reproductive technologies; **AUC**, area under the curve; **BCS**, body condition score; **BRD**, bovine respiratory disease; **BTW**, birthweight; **CP**, crude protein; **CRL**, crown rump length; **ET**, embryo transsfer; **FSH**, follicle-stimulating hormone; **GH**, growth hormone; **GHR**, growth hormone receptor; **GnRH**, gonadotrophin releasing hormone; **GTT**, glucose tolerance test; **HBW**, high birthweight; **HPA,** hypothalamic-pituitary-adrenal axis; **HPG**, hypothalamic-pituitary-gonadal axis; **IGF**, insulin-like growth factor; **IGFBP**, insulin-like growth factor binding protein; **IVP**, in vitro embryo production; **IUGR**, intra-uterine growth retardation; **LBW**, low birthweight; **LH**, luteinizing hormone; **MOET**, multiple-ovulation embryo transfer; **MP**, multiparous; **NP**, nulliparous; **PCOS**, polycystic ovary syndrome; **PP**, primiparous; **S/C,** services per conception; **SGA**, small for gestational age; **THI**, temperature humidity index.
